# Relationship between body mass index, gray matter volume and peripheral inflammation in patients with post-COVID condition

**DOI:** 10.1016/j.bbih.2025.101137

**Published:** 2025-11-05

**Authors:** Luise Victoria Claaß, Franziska Schick, Tonia Rocktäschel, Alejandra P. Garza, Christian Gaser, Philipp A. Reuken, Andreas Stallmach, Kathrin Finke, Sharmili Edwin Thanarajah, Martin Walter, Ildiko Rita Dunay, Bianca Besteher, Nils Opel

**Affiliations:** a Charite - Universitatsmedizin Berlin, Department of Psychiatry and Neurosciences, Campus Benjamin Franklin, Berlin, Germany; b DZPG, German Center for Mental Health, site Berlin, Germany; c German Center for Mental Health (DZPG), Germany; d Center for Intervention and Research on adaptive and maladaptive brain Circuits underlying mental health (C-I-R-C), Jena-Magdeburg-Halle, Germany; e Institute of Inflammation and Neurodegeneration, Otto-von-Guericke University Magdeburg, Magdeburg, Germany; f Medical Physics Group, Institute of Diagnostic and Interventional Radiology, Jena University Hospital - Friedrich Schiller University Jena, Germany; g Department of Internal Medicine IV, Gastroenterology, Hepatology and Infectious diseases, Jena University Hospital, Germany; h Department of Neurology, Jena University Hospital, Germany; i Center of Sepsis Control and Care, Jena University Hospital, Germany; j Department of Psychology, Ludwig-Maximilians-Universitaet München, Germany; k Max Planck Institute for Metabolism Research, Cologne, Germany; l Department of Psychiatry, Psychosomatic Medicine and Psychotherapy, University Hospital Frankfurt, Frankfurt, Germany; m Fraunhofer Institute for Translational Medicine and Pharmacology, Frankfurt, Germany

## Abstract

**Background:**

Obesity, a condition associated with low-grade peripheral inflammation, is an independent risk factor for severe COVID-19 and has been linked to structural brain alterations. Given that post-COVID condition (PCC) is also associated with structural brain abnormalities and lingering immunological alterations, this study aimed to assess whether obesity contributes to these neural and immunological differences in PCC patients.

**Methods:**

We investigated a previously established cohort of PCC patients (n = 61), recruited between April 2021 and June 2022. Whole-brain comparison of gray matter volume (GMV) was conducted by voxel-based morphometry (VBM). Obesity, as measured by body mass index (BMI), as well as age, sex, and total intracranial volume (TIV), were included as regressors in a linear model. Signature immunological markers were quantified in 50 participants using a LEGENDplex™ multiplex bead-based assay.

**Results:**

A significant negative association was found between BMI and GMV in the right thalamus (p(FWE) = 0.039, k = 209, TFCE = 1037.97, x = 18, y = −21, z = 8). Moreover, BMI and thalamic GMV were significantly associated with immunological markers in PCC. Specifically, BMI was positively associated with Interleukin-6 (p = 0.021) and negatively with Interleukin-7 (p = 0.021), while GMV showed positive associations with Interleukin-8 (p = 0.05).

**Conclusion:**

The results suggest that BMI contributes to GMV alterations in PCC patients, with both BMI and GMV demonstrating correlations with peripheral immunological markers. These findings indicate that converging mechanisms involving inflammation and structural brain alterations may contribute to obesity and PCC.

## Highlights

• Higher body mass index is linked to lower gray matter volume in post-COVID patients.
• Brain structural alterations and body mass index link to immunological changes.
• These changes are most pronounced in patients with cognitive deficits.
• Obesity and post-COVID condition may share converging pathomechanisms.

## 1 Introduction

Elevated body mass index (BMI), as an indicator of obesity, has been extensively associated with metabolic diseases and systemic, low-grade inflammation (Hotamisligil, 2017). Key mechanisms likely include the production of proinflammatory immunological markers and hormonal activity by adipocytes, perturbed glucose metabolism with insulin resistance, dyslipidemia, inflammation-associated endothelial dysfunction, and tissue hypoxia (Engin, 2017; Hotamisligil, 2017; Hotamisligil et al., 1993; Lefere et al., 2016; Uysal et al., 1997; Vekic et al., 2019; Wang et al., 2023). Furthermore, the significance and implications of obesity as one of the most prevalent somatic comorbidities of psychiatric conditions, such as major depressive disorder (MDD), have received more recognition (de Wit et al., 2010). From this perspective, the relationship between higher BMI and structural brain alterations has been the focus of extensive research. In this context, patterns of cortical thickness reduction similar to those observed in various neuropsychiatric disorders, such as MDD and bipolar disorder, have been found in obesity (Opel et al., 2021).

Upon the beginning of the pandemic caused by severe acute respiratory syndrome coronavirus 2 (SARS-CoV-2), obesity was identified as a major risk factor for severe outcomes of the resulting disease, coronavirus disease 2019 (COVID-19) (Docherty et al., 2020; Petrilli et al., 2020). This risk is likely due to systemic chronic inflammation, dysregulated metabolism, a dysfunctional immune system, an inflamed endothelium, impaired mesenchymal stromal cells, and altered adipose tissue (Ritter et al., 2020). A previous study demonstrated that SARS-CoV-2 invades human adipose tissue by targeting both mature adipocytes and a subset of adipose tissue macrophages, which leads to immune system activation and the release of immunological markers associated with severe cases of COVID-19 (Martínez-Colón et al., 2022).

Correspondingly, elevated pro-inflammatory immunological markers such as Interleukin (IL)-1β, Interleukin-6, and Tumor Necrosis Factor-alpha (TNF-α) have been linked to obesity (Hotamisligil, 2017) as well as to post-COVID condition (PCC), likely produced by overactivated monocytes and macrophages (Schultheiss et al., 2022). Additionally, PCC has been linked to structural brain alterations. For instance, a large-scale longitudinal study by Douaud et al. found a reduction in gray matter thickness in the orbitofrontal cortex and parahippocampal gyrus (Douaud et al., 2022). Moreover, abnormalities in diffusion measures, which indicate tissue damage, were observed in regions functionally connected to the primary olfactory cortex. Patients with SARS-CoV-2 infection also exhibited a greater reduction in overall brain size (Douaud et al., 2022). In contrast, other preceding studies found wide-spanning increases in gray matter volume (GMV), alongside lingering immunological shifts, most pronounced in PCC patients with cognitive deficits (Besteher et al., 2022, 2024).

In sum, preliminary evidence suggests that both obesity and the long-term consequences of COVID-19 show converging effects on cortical alterations and peripheral low-grade inflammation.

To the best of our knowledge, there has been no comprehensive investigation of BMI as a modulating factor on immunological markers and structural brain alterations in patients with post-acute COVID-19 sequelae yet. Since obesity is a preventable risk factor, understanding its exact impact on PCC progression would be valuable. Identifying increases in peripheral inflammation and cortical thickness alterations could explain how obesity contributes to more severe PCC courses. Additionally, we plan to include exploratory analyses to gain insights specifically into patients experiencing more severe functional consequences due to brain alterations in regions critical for neurocognitive functions. Our goal is to characterize the sources of interindividual variability in PCC by integrating clinical, neuroimaging, and immunological data.

## 2 Methods

### 2.1 Participants

The study participants are part of a previously established clinical cohort (Besteher et al., 2024). The study included 61 patients with PCC, recruited between April 2021 and June 2022 from the post-COVID outpatient clinic of the Department of Internal Medicine IV (Infectiology) and the Department of Neurology at Jena University Hospital.

As in the previously published report on this cohort (Besteher et al., 2024), to examine a subpopulation with cognitive deficits in more detail, we divided the patients into two subgroups based on the results of the neurocognitive screening with the Montreal Cognitive Assessment (MoCA), using a pre-specified cutoff of below 26 (mild to moderate cognitive impairment) and 26 or higher (no cognitive impairment) (Malek-Ahmadi et al., 2014; Nasreddine et al., 2005).

For the final cohort, the demographic characteristics are described in Table 1.

**Table 1:** Demographic characteristics of the PCC cohort.

	n
Total	61
MoCA ≥26	35
MoCA <26	26
Sex	37 female (60.7 %)

	Mean (SD)	Range (min – max)
Age (years)	45.26 (12.93)	20–73
BMI (kg/m^2^)	26.11 (4.40)	18.21–36.13
Years of education	11.12 (1.03)	9–12
Time since infection (months)	9.94 (4.04)	1–24.5

SD = standard deviation, BMI = body mass index, MoCA = Montreal Cognitive Assessment.

Initially, in each participant, the acute SARS-CoV-2 infection was detected using real-time reverse transcriptase polymerase chain reaction (RT-qPCR) at the time of acute infection. When visiting the post-COVID outpatient clinic, this test result was confirmed, and a licensed physician took a medical history, including the timing and severity of COVID-19 symptoms according to WHO guidelines.

**Fig. 1:**
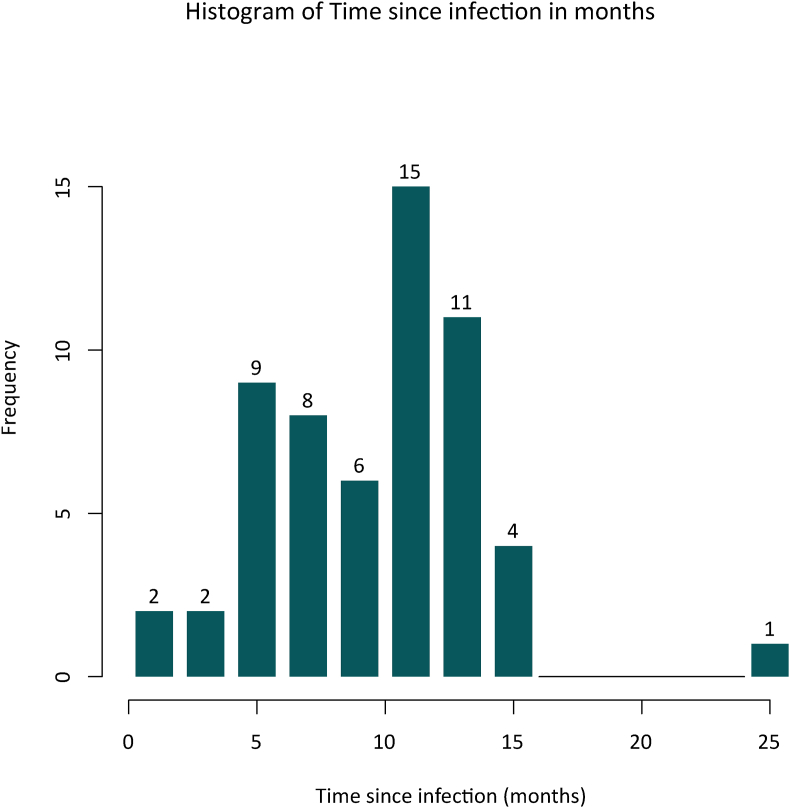
Frequency distribution of time since infection in months. Numbers above the bars are absolute counts of patients.

There were no restrictions on inclusion criteria concerning specific symptoms or the time elapsed since infection, allowing for coverage of the full spectrum of post-acute sequelae of COVID-19. The distribution of time since infection within our cohort is shown in Fig. 1. Participants had no history of psychiatric conditions, as confirmed by thorough screening using the MINI interview conducted by trained personnel. Additionally, none of the participants had a history of severe neurological disorders, untreated relevant medical conditions, acute infections treated with antibiotics, substance abuse, or psychiatric disorders among first-degree relatives. All participants met the inclusion criteria for MRI examination and provided written informed consent to take part in the study.

The study protocol was approved by the local Ethics Committee of the Medical Faculty of the University of Jena.

### 2.2 Magnetic resonance imaging

Participants underwent a high-resolution T1-weighted magnetic resonance imaging (MRI) scan on a 3 T S Prism Fit scanner (Siemens, Erlangen, Germany). The scan was conducted using a standard quadrature head coil and an axial 3-dimensional magnetization prepared rapid gradient echo (MP-RAGE) sequence, with the following parameters: TR 2400 ms, TE 2.22 ms, α 8°, 208 contiguous sagittal slices, FoV 256 mm, voxel resolution 0.8 × 0.8 × 0.8 mm. The acquisition took 6 min and 38 s. Before the native brain MRI, all participants gave informed consent to the radiological examination after consulting with a licensed radiologist. This scan was part of a 60-min MRI protocol. All images were checked to ensure that no artifacts were present.

### 2.3 Voxel-based morphometry

Voxel-based morphometry (VBM) was performed using the CAT12 toolbox, developed by the Structural Brain Mapping group at Jena University Hospital in Jena, Germany (Gaser et al., 2023). This toolbox is integrated into the SPM12 software, produced by the Institute of Neurology in London, UK. All T1-weighted images underwent bias field correction. Subsequently, the images were segmented into gray matter (GM), white matter (WM), and cerebrospinal fluid (CSF) (Ashburner and Friston, 2005). The images were spatially normalized using the DARTEL algorithm (Ashburner, 2007). The segmentation process was extended to include adaptive maximum a posteriori estimation (Rajapakse et al., 1997) and to address partial volume effects (Tohka et al., 2004). After pre-processing, an automated quality control protocol was performed to ensure the absence of artifacts. Next, all scans were smoothed with an 8 mm (Full Width at Half Maximum) Gaussian kernel. To focus the analysis on GM areas, an absolute GM threshold of 0.2 was applied. Data homogeneity and orthogonality were verified, and outliers with a mean absolute z-score exceeding 2 standard deviations were excluded.

### 2.4 Blood collection and serum levels of immunological markers

For the assessment of immunological markers, all participants underwent standardized blood collection within the designated time frame between 7:30 and 9:00 a.m. on the day of the MRI scan. Participants were not fasted. 7.5 mL serum gel monovettes (Sarstedt) were used, and 30 min after extraction, centrifugation was carried out at 1800 g for 10 min. The samples were divided into aliquots of 0.25 mL each and frozen at −80 °C. Frozen samples were then transferred to our cooperating institute in Magdeburg, where serum was thawed at 4 °C, followed by a centrifugation step at 400 g for 10 min to remove any debris. The quantification of multiple key immunological markers, including IL-10, IFN-γ, IL-6, TNF-α, CXCL10, sTREM2, sTREM-1, CCL2 (MCP-1), IL-18, BDNF, VEGF, β-NGF, sRAGE, CX3CL1, α-synuclein, G-CSF, IFNα2, IL-2, IL-7, IL-1RA, and CXCL8, was performed using the LEGENDplex™ multiplex bead-based assay (BioLegend, 741091, 740795, 741197) following the manufacturer's instructions as previously described (Garza et al., 2023).

### 2.5 Statistical analysis

#### 2.5.1 VBM

One outlier in the VBM analysis was excluded from further analysis. A multiple linear regression model, implemented in CAT12, was used to examine the association between BMI and whole-brain GMV across the entire cohort. BMI, age, and sex were included as covariates in the model to account for related variance in all subsequent analyses. Total intracranial volume (TIV), as an indicator of head size, was also included as a covariate, since there was no significant correlation between BMI and TIV in this patient group. An absolute threshold of 0.2 was used for masking. To examine whether the relationship between BMI and GMV varied based on cognitive status, we tested the interaction term (BMI × MoCA-Group) using a second whole-brain multiple regression model. The results from the VBM were corrected using threshold-free cluster enhancement (TFCE) (Spisák et al., 2019) with 5000 permutations and then corrected for multiple comparisons using the family-wise error (FWE) method, with a significance level of *p* < 0.05. The anatomical labelling of clusters was conducted in accordance with the AAL atlas (Tzourio-Mazoyer et al., 2002).

#### 2.5.2 Immunological markers

Statistical analysis and data visualization of serum immunological markers were performed using R. Outliers were identified through z-scores with a significance threshold of 0.05 and visual inspection of scatterplots. The data distribution was non-normal, as indicated by Shapiro-Wilk tests. Therefore, Spearman's rank correlation test was used to evaluate the association between all immunological markers and both BMI and GMV values, to determine if a linear relationship existed between BMI and specific immunological markers. To further assess how well BMI or GMV could be explained by particular immunological variables while adjusting for age and sex, multiple linear regression analyses were conducted. Next, interaction terms between each immunological marker and the MoCA group were added to the model to examine whether the relationships between immunological markers and BMI or GMV varied with cognitive status, thus testing for group-dependent effects. Finally, Spearman's rank correlation tests were applied to analyze associations between immunological markers and BMI, as well as GMV, in the two subgroups based on MoCA scores, separately. All statistical tests were performed with an alpha level of *p* < 0.05, and *p*-values of 0.05 or less were considered statistically significant. For multiple comparisons, we controlled the false discovery rate at 0.05 using the Benjamini-Hochberg procedure (Benjamini and Hochberg, 1995).

## 3 Results

### 3.1 VBM

**Fig. 2:**
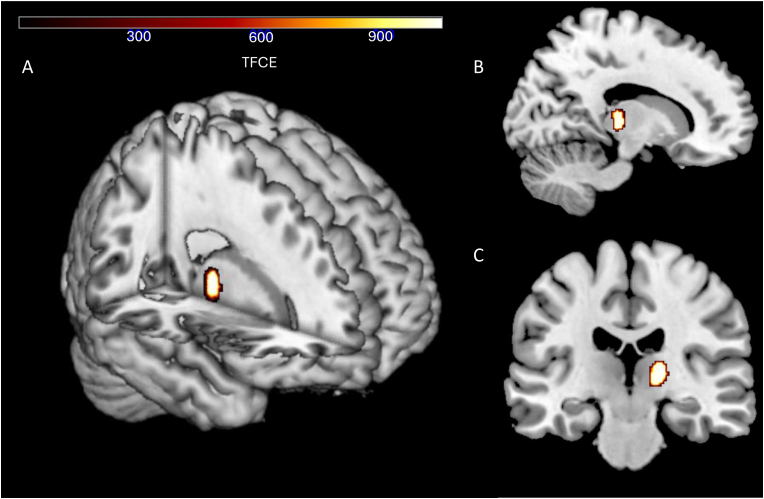
Reduced gray matter volume in patients with post-COVID condition correlates with BMI. Significant cluster in right thalamus (peak at MNI coordinates = 18–21 8, p(FWE) = 0.039) is presented as an overlay. **A** 3D Render visualization of the peak cluster. **B** Sagittal slice at x = 14, y = −27, z = 35. **C** Coronal slice at x = 34, y = −22, z = 26.

**Fig. 3:**
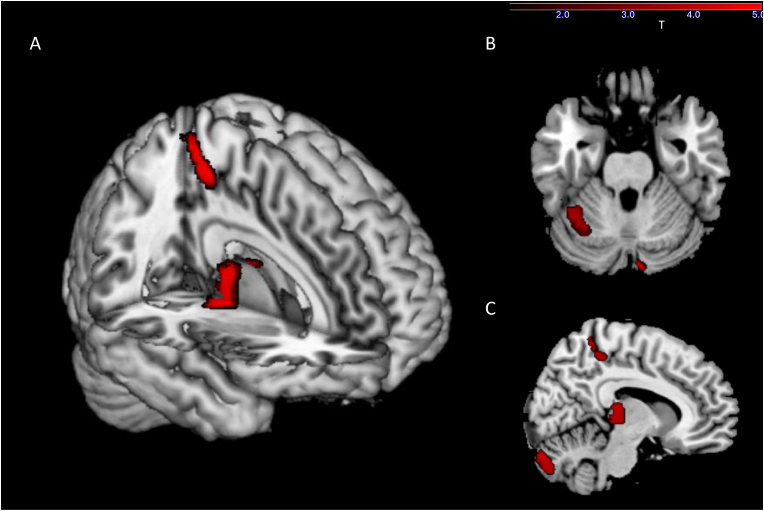
Exploratory sensitivity analyses employing an uncorrected threshold of *p* < 0.001 and a minimal cluster of k = 100 showed several clusters with GMV reduction in relation to higher BMI. **A** 3D Render visualization. **B** Axial slice at MNI coordinates x = 3, y = 15, z = −26. **C** Sagittal slice at x = 9, y = 3, z = 32.

The multiple linear regression showed a correlation between BMI and a decrease in GMV, with a significant cluster in the right thalamus (p(FWE) = 0.039, k = 209, TFCE = 1037.97, x = 18, y = −21, z = 8), as illustrated in Fig. 2. Exploratory sensitivity analyses using an uncorrected threshold of *p* < 0.001 and a minimum cluster size of k = 100 identified several widespread clusters with GMV differences related to BMI. These clusters included regions such as the left lobule IV of the cerebellar hemisphere and the right inferior parietal gyrus, excluding the supramarginal and angular gyri. A 3D Render visualization of these clusters is shown in Fig. 3, estimates and coordinates are listed in Table S1 in the Supplement. No correlation between BMI and an increase in GMV was found, neither at pFWE <0.05 nor at the uncorrected exploratory threshold.

In additional analyses to specifically examine a subgroup with cognitive deficits, we divided the patient cohort into two groups. The subset with reduced cognitive functioning comprised 26 patients (mean MoCA value 23.84, SD = 1.43), and the subgroup with normal cognitive functioning comprised 35 patients (mean MoCA value 27.45, SD = 1.48).

A second whole-brain VBM analysis revealed no significant interaction between BMI and cognitive status (MoCA group) on GMV, indicating that the presence of cognitive deficits did not strongly moderate the association between BMI and GMV.

### 3.2 Immunological markers

Data on peripheral immunological markers were available for 50 of the 60 PCC patients included in the VBM analysis. Outlier detection resulted in the exclusion of 2 subjects from the dataset.

#### 3.2.1 Immunological markers and BMI

A multiple linear regression model with BMI as the dependent variable and immunological markers, age, and sex as covariates showed a significant effect (F(24,15) = 2.65, p = 0.027), indicating a positive relationship between BMI and IL-6 (p = 0.021) and a negative relationship with IL-7 (p = 0.021). A sensitivity analysis of IL-6 and IL-7 showed no significant relationship with time since infection, respectively.

No significant interaction was observed between immunological markers and cognitive status (MoCA group) in linear regression models predicting BMI. Estimates and standard errors of the regression analysis are shown in Table S2 in the supplement, and results of the interaction analysis are displayed in Table S3 in the supplement.

#### 3.2.2 Immunological markers and GMV

To further investigate the relationship between immunological marker levels and brain structure, we extracted GMV data using the eigenvariate function in SPM12. The eigenvariate values of the cluster in the right thalamus showed a positive correlation with IL-8 (p = 0.050). However, this correlation did not remain significant after adjusting for multiple comparisons using the Benjamini-Hochberg procedure. Results of the correlation analysis between GMV and immunological markers can be found in Table S4 in the supplement.

Next, a linear regression analysis with GMV as the dependent variable and immunological markers, age, and sex as covariates was performed, which resulted in no significant findings. When including the presence of cognitive deficits (MoCA group) as a covariate in this linear regression model, the interaction between sTREM-1 and cognitive status (MoCA group) was statistically significant (p(FDR) = 0.015). This indicates that the relationship between sTREM-1 levels and GMV in the thalamic cluster varies depending on cognitive performance. Specifically, in individuals with preserved cognition (MoCA score ≥26), higher sTREM-1 levels correlated with lower GMV, while in cognitively impaired participants (MoCA score <26), this relationship was reversed, suggesting a group-specific modulation of neuroinflammatory effects on GMV. Results of the interaction analysis is shown in Table S5 in the supplement.

#### 3.2.3 Comparison of BMI and GMV in relation to immunological markers

**Fig. 4:**
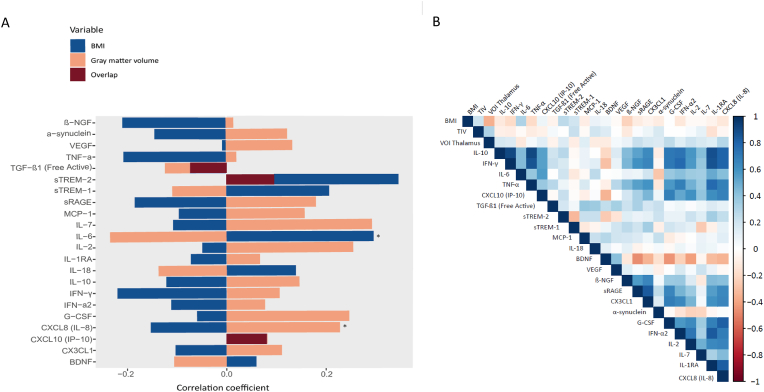
**A** Correlation coefficients between immunological markers and BMI, as well as between immunological markers and eigenvariate values of the cluster associated with GMV reduction in patients with higher BMI. **B** Heatmap of the correlation coefficients of BMI, total intracranial volume (TIV), eigenvariate values of the cluster in the right thalamus (VOI_Thalamus), and immunological markers.

Fig. 4A visualizes the correlation coefficients between immunological markers and BMI and between immunological markers and GMV. Overall, the direction of the association differs notably between the two outcomes: while most immunological markers are positively associated with GMV reduction in the right thalamus, BMI has a negative association with many immunological markers. This difference is not surprising, as the GMV measure itself was inversely related to higher BMI. Only three markers showed consistent effects for BMI and GMV reduction: TGF-1, sTREM-2, and IP-10/CXCL10.

Fig. 4B shows the correlation matrix between BMI, GMV, TIV, and immunological markers. In addition to the associations shown in Fig. 4A, it also displays the correlation strengths among the immunological markers themselves. Strong positive correlations are observed among several immunological markers. Conversely, BDNF shows generally more negative associations with immunological markers and with GMV, while being positively correlated with BMI.

#### 3.2.4 Comparison of subgroups based on cognitive function

To further explore potential differences between the two subgroups based on cognitive performance, correlations were examined separately in each group. This analysis revealed varying relationships among immunological markers, BMI, and GMV. In participants with reduced cognitive function, BMI was positively linked to IL-6 and negatively linked to Interferon-γ and TNF-α. However, after adjusting for multiple comparisons, only a trend-level effect for IL-6 remained. In this patient group, GMV showed a significant positive correlation with IL-2 and IL-8.

In participants with normal cognitive functioning, BMI and GMV were both differently associated with sTREM-1, although these effects did not remain significant after correction for multiple testing. All correlation coefficients with confidence intervals and p-values are provided in Table S6–S9 in the supplement.

## 4 Discussion

In the present study, we found an association between BMI and GMV in PCC patients, with the most pronounced GMV alterations in the thalamus. Moreover, GMV alterations were in turn associated with peripheral immune markers, but only in the patient group with impaired cognitive function. This might indicate involvement of an immunometabolic pathway interconnected with these alterations. These findings thus point to the mutual interaction between higher BMI, systemic inflammation (or immune activation), and brain structural abnormalities in PCC.

First, it appears relevant to take into account previous literature on BMI- and obesity-related brain structural alterations. In this regard, a meta-analysis pooling data from 10 studies using whole-brain VBM analysis found that several GM areas were decreased in size, most pronounced in frontal, temporal, insular, and cerebellar regions. Additionally, the authors identified some clusters with increased GMV in patients with obesity, with the largest cluster located in the cuneus (Herrmann et al., 2019). Similarly, García-García et al. reported that obesity and body mass are linked to significantly lower GMV across various areas crucial for executive control, including the medial prefrontal cortex, bilateral cerebellum, and left temporal pole (García-García et al., 2019). Furthermore, widespread BMI-related GMV alterations were observed in both healthy and neuropsychiatric populations (Opel et al., 2021).

While our finding of GMV reductions aligns with previous findings, the pattern of regional distribution differs from most previous studies. Future replication studies are needed to determine potential underlying reasons, such as the study population or comorbidities, that may contribute to these differences.

Apart from considering previous finings on obesity, it appears relevant to consider the functional role of the thalamus: The thalamus, particularly the paraventricular thalamic nucleus (PVT), has been linked to homeostasis of food intake and feeding behavior (Millan et al., 2017) as well as stress regulation (Hsu et al., 2014), making it plausible that its structure could be altered in obesity. In fact, previous volumetric imaging studies have shown increased thalamic volumes in obesity (Bernardes et al., 2018; Opel et al., 2021), whereas other studies using VBM found an inverse relationship between BMI or waist circumference and thalamic GMV, respectively (Janowitz et al., 2015; Kharabian Masouleh et al., 2016). This emphasizes the importance of distinguishing between measures of GMV and density and underscores the difficulty of comparing GM measurements in obesity across different methods (Perlaki et al., 2018). In the UK Biobank study examining brain structure differences before and after an acute SARS-CoV-2 infection, the most notable effect was seen in thalamic volume when only cross-sectional comparisons between the groups at the second post-infection time point were performed (Douaud et al., 2022). However, when baseline scans were included, this effect was no longer present, as the thalamus of participants who later contracted the infection appeared different from that of healthy controls years earlier. Therefore, while the influence of PCC on thalamic GMV remains uncertain in current research, BMI has consistently been linked to structural alterations in this region.

The present study furthermore revealed associations of BMI with peripheral immune markers. We observed a significant positive association between BMI and IL-6, which was independent of the time since infection. In the general population, elevated IL-6 levels have frequently been linked to obesity and metabolic dysfunction (Ouchi et al., 2011). In our PCC cohort, when divided based on the MoCA score as an indicator of cognitive functioning, BMI showed a trendwise positive correlation with IL-6 only in the group with reduced cognitive ability. This aligns with previous research connecting high IL-6 levels to disrupted circuits involved in learning and memory (Jankowsky and Patterson, 1999). Potential mechanisms discussed in current literature that may underlie the link between IL-6 levels and cognitive deficits include the neurodegenerative effects of elevated IL-6 levels (Harrell et al., 2021; Morales et al., 2010) and its association with blood-brain barrier dysfunction and subsequent cognitive impairment (Mei et al., 2021). In relation to PCC, IL-6 has been suggested as a potential mediator of long-term neuropsychiatric symptoms of COVID-19 (Kappelmann et al., 2021).

Examining the relationship between IL-6 and body weight more closely, Milaneschi et al. found that higher IL-6 levels correlated with decreased appetite (Milaneschi et al., 2021). Recent meta-analytic results suggested a link between the IL-6 pathway and weight regulation, with IL-6 pathway inhibitors associated with BMI increases (Patsalos et al., 2020). Together with Glucagon-like peptide 1 (GLP-1), it is thought to control weight in the gut and brain via central and peripheral signaling (Yang et al., 2025).

Overall, elevated IL-6 levels appear to be consistently associated with higher BMI and may play a role in neurocognitive impairment; however, their complex involvement in appetite regulation highlights the multifaceted effects of this cytokine.

Furthermore, when linking brain structural imaging with immunological markers, we observed a correlation between BMI-related GMV alterations and IL-8 and IL-2, respectively, both of which have previously been linked to COVID-19 severity and prognosis (Li et al., 2020; Shi et al., 2020; Torres-Ruiz et al., 2022). Notably, this association was only evident in the group with impaired cognitive functioning. Although IL-6 was not directly associated with GMV reduction, its association with BMI suggests an indirect link. Relatedly, a recent study reported a correlation between elevated IL-6 levels and reduced cortical and subcortical brain volumes—including the thalamus—with effects especially prominent in the right hemisphere (Zhao et al., 2024). In the same study, higher IL-6 levels were again associated with poorer performance on cognitive tests.

Our findings indicate that BMI related thalamic volume alterations are connected to immunological markers also associated with more severe COVID-19 outcomes, and this connection was stronger in patients with cognitive deficits. This may support the idea that obesity and PCC share or mutually reinforce neuroinflammatory mechanisms contributing to neurocognitive dysfunction.

Nonetheless, the practical implications of our findings are not yet fully decoded. In COVID-19, elevated IL-6 levels are linked to greater disease severity (Queiroz et al., 2022). IL-6 inhibitors such as tocilizumab have demonstrated protective effects in early stages of COVID-19 against later development of depressive symptoms (Benedetti et al., 2021). The first anecdotal evidence for using tocilizumab to treat neurocognitive symptoms of PCC has emerged, but more research is needed to confirm its efficacy and side effects (Visvabharathy et al., 2022). Of note, IL-6 inhibitors have also been associated with an increase in BMI when used to treat immune disorders, and, in general populations, higher IL-6 levels appear to reduce appetite and regulate weight (Milaneschi et al., 2021; Patsalos et al., 2020; Yang et al., 2025).

It is essential to recognize that while the current findings enhance our understanding of the underlying mechanisms, they do not allow for direct conclusions about treatment options. Ongoing clinical studies are necessary to explore potential translational implications. In this context, anti-inflammatory and immunomodulatory therapies—such as Janus Kinase inhibitors like upadacitinib—are currently under investigation (ClinicalTrials.gov ID: NCT06928272). A study analyzing biomarker profiles in PCC patients who reported symptom improvement after extracorporeal therapeutic apheresis observed a decrease in autoantibodies, lipids, and inflammation markers, including IL-1beta, IL-6, and CRP (Achleitner et al., 2023).

Thus, anti-inflammatory treatment could not only benefit the course of severe COVID-19 and post-COVID conditions but also positively affect obesity-related low-grade inflammation and its adverse effects. However, there may also be a risk of worsening obesity during treatment with cytokine inhibitors like tocilizumab. Beyond the context of PCC, the potential of anti-inflammatory strategies has also been emphasized in psychiatric conditions: recent meta-analyses revealed that adjunctive anti-inflammatory treatment can reduce depressive symptoms in patients with MDD (Du et al., 2024; Kappelmann et al., 2018).

## 5 Limitations

A limitation of the study is the small cohort size and the absence of data on BMI changes before and after SARS-CoV-2 infection, as this information would help us understand possible weight loss or gain related to the infection. Given the cross-sectional design of the current study, conclusions regarding causal relationships must be drawn with utmost caution. To explore potential bidirectional relationships among BMI, structural brain alterations, and inflammation, future research should use longitudinal designs and mediation analyses. Additionally, we did not measure obesity-related markers such as insulin, C-peptide, glucagon, leptin, or cortisol. The applicability of our findings may be limited because the study sample was drawn solely from a European clinical population. Sociocultural factors, healthcare system differences, and environmental influences unique to each region could affect both clinical symptoms and biological markers. Therefore, caution is advised when applying these findings to populations outside of Europe.

## 6 Conclusion

We demonstrated not only a reduction in GMV associated with higher BMI in a cohort of PCC patients but also connected these abnormalities, along with BMI, to distinct immunological shifts, most pronounced in patients with cognitive deficits. This may help clarify potential shared mechanisms of obesity and PCC related to chronic inflammation and structural brain alterations. Going forward, more research in this area could lead to the development of targeted immunomodulatory treatments for PCC in obese patients.

## CRediT authorship contribution statement

**Luise Victoria Claaß:** Writing – review & editing, Writing – original draft, Visualization, Project administration, Methodology, Investigation, Formal analysis, Data curation, Conceptualization. **Franziska Schick:** Writing – review & editing, Project administration, Investigation, Formal analysis, Data curation. **Tonia Rocktäschel:** Writing – review & editing, Methodology, Investigation, Formal analysis, Data curation, Conceptualization. **Alejandra P. Garza:** Writing – review & editing, Writing – original draft, Visualization, Methodology, Investigation, Formal analysis, Data curation, Conceptualization. **Christian Gaser:** Writing – review & editing, Software, Resources, Methodology, Investigation. **Philipp A. Reuken:** Writing – review & editing, Supervision, Resources, Investigation, Conceptualization. **Andreas Stallmach:** Writing – review & editing, Supervision, Resources, Investigation, Conceptualization. **Kathrin Finke:** Writing – review & editing, Supervision, Methodology, Investigation, Conceptualization. **Sharmili Edwin Thanarajah:** Writing – review & editing, Supervision, Methodology, Investigation. **Martin Walter:** Writing – review & editing, Supervision, Resources, Investigation, Conceptualization. **Ildiko Rita Dunay:** Writing – review & editing, Supervision, Resources, Methodology, Investigation, Conceptualization. **Bianca Besteher:** Writing – review & editing, Writing – original draft, Supervision, Methodology, Investigation, Funding acquisition, Conceptualization. **Nils Opel:** Writing – review & editing, Writing – original draft, Visualization, Supervision, Resources, Methodology, Investigation, Funding acquisition, Conceptualization.

## Declaration of generative AI and AI-assisted technologies in the manuscript preparation process

During the preparation of this work the authors used Grammarly in order to improve language and readability. After using this tool, the authors reviewed and edited the content as needed and take full responsibility for the content of the published article.

## Funding

SET was funded by the Leistungszentrum Innovative Therapeutics (TheraNova), funded by the 10.13039/501100003185Fraunhofer Society and the Hessian Ministry of Science and Art, the Bundesministerium für Bildung und Forschung (10.13039/501100002347BMBF, Federal Ministry of Education)- 01EO2102 INITIALISE Advanced Clinician Scientist Program, and the REISS foundation.

## Declaration of competing interest

The authors declare the following financial interests/personal relationships which may be considered as potential competing interests:Sharmili Edwin Thanarajah reports financial support was provided by Leistungszentrum Innovative Therapeutics funded by the 10.13039/501100003185Fraunhofer Society and the Hessian Ministry of Science and Art, 10.13039/501100002347BMBF (Federal Ministry of Education) and the REISS foundation. If there are other authors, they declare that they have no known competing financial interests or personal relationships that could have appeared to influence the work reported in this paper.

## Data Availability

Data will be made available on request.
