# Supplementary material for: Relationship between body mass index, gray matter volume and peripheral inflammation in patients with post-COVID condition
Source: Brain Behav Immun Health. 2025 Nov 5;50:101137. doi: 10.1016/j.bbih.2025.101137 (PMC12663082; doi:10.1016/j.bbih.2025.101137)
Supplement: Multimedia component 1 [file mmc1.docx]

**Supplementary Material**

**Journal:** Brain, Behavior, & Immunity - Health

**Relationship between Body Mass Index, Gray Matter Volume and peripheral inflammation in patients with post-COVID condition**

Luise V. Claaß, Franziska Schick, Tonia Rocktäschel, Alejandra P. Garza, Christian Gaser, Philipp A. Reuken, Andreas Stallmach, Kathrin Finke, Sharmili Edwin Thanarajah, Martin Walter, Ildiko Rita Dunay, Bianca Besteher#, Nils Opel#

# This is to indicate that both authors contributed equally and should therefore both be considered senior author

**Corresponding author:** N. Opel, Department of Psychiatry and Psychotherapy, Jena University Hospital/Friedrich-Schiller-University Jena, Philosophenweg 3, 07743 Jena, Germany, Phone: +49 36419390101, Email: nils.opel@med.uni-jena.de

| Anatomical region | T | Z | Cohen's d | k | p(FWE) | TFCE | Coordinates |
| --- | --- | --- | --- | --- | --- | --- | --- |
| Right Ventral posterolateral (Thalamus).  Right Pulvinar medial | 4.40 | 4.06 | 1.187 | 209 | 0.039 | 1037.97 | 18 -21 8 |
| Left Lobule IV of cerebellar hemisphere | 3.92 | 3.67 | 1.057 | 1.343 | 0.060 | 936.20 | -30 -64 -22 |
| Right Inferior parietal gyrus -  excluding supramarginal and angular gyri |  |  |  | 106 | 0.312 | 521.74 | 52 -51 46 |

Table S1. Estimates of the exploratory clusters in the VBM analysis.

Table S2. Linear regression analysis on the association of BMI with cytokines, age and gender.

| Immunological Marker | Estimate | Standard Error | t-value | p-value |
| --- | --- | --- | --- | --- |
| IL-10 | 0.38 | 0.33 | 1.15 | 0.267 |
| IFN-γ | -0.14 | 0.08 | -1.87 | 0.081 |
| IL-6 | 0.79 | 0.31 | 2.58 | 0.021 |
| TNF-α | 0.01 | 0.08 | 0.14 | 0.894 |
| CXCL10 (IP-10) | 0.01 | 0.02 | 0.69 | 0.499 |
| TGF-β1 (Free Active) | -0.01 | 0.02 | -0.52 | 0.612 |
| sTREM-2 | 0.01 | 0.01 | 1.25 | 0.231 |
| sTREM-1 | 0.01 | 0.02 | 0.59 | 0.563 |
| MCP-1 | -0.06 | 0.05 | -1.21 | 0.245 |
| IL-18 | 0.02 | 0.02 | 0.99 | 0.339 |
| BDNF | 0.00 | 0.00 | -0.91 | 0.375 |
| VEGF | 0.01 | 0.03 | 0.39 | 0.701 |
| β-NGF | -0.19 | 0.14 | -1.36 | 0.193 |
| sRAGE | 0.02 | 0.01 | 2.00 | 0.064 |
| CX3CL1 | -0.01 | 0.00 | -1.95 | 0.070 |
| α-synuclein | 0.00 | 0.00 | -1.90 | 0.077 |
| G-CSF | 0.02 | 0.03 | 0.50 | 0.623 |
| IFN-α2 | 0.98 | 0.82 | 1.20 | 0.247 |
| IL-2 | -0.81 | 0.69 | -1.16 | 0.263 |
| IL-7 | -0.33 | 0.13 | -2.57 | 0.021 |
| IL-1RA | -0.11 | 0.10 | -1.09 | 0.292 |
| CXCL8 (IL-8) | 0.10 | 0.16 | 0.64 | 0.531 |
| sex | 3.38 | 1.87 | 1.80 | 0.092 |
| age | 0.04 | 0.09 | 0.51 | 0.615 |

Table S3. Results of linear regression models testing the interaction between immunological markers and cognitive status on BMI. The β coefficient for the interaction term represents the change in slope of the association between the respective marker and BMI in the cognitively normal group (MoCA ≥ 26) compared to the impaired group (MoCA < 26).

| Immunological  Marker | β (Interaction  Estimate) | Standard Error | t-value | p-value | p(FDR) |
| --- | --- | --- | --- | --- | --- |
| IL-10 | 0.35 | 0.18 | 1.93 | 0.060 | 0.302 |
| IFN-γ | 0.09 | 0.04 | 2.05 | 0.047 | 0.302 |
| IL-6 | -0.50 | 0.34 | -1.45 | 0.155 | 0.581 |
| TNF-α | 0.10 | 0.04 | 2.26 | 0.029 | 0.302 |
| sTREM-2 | 0.01 | 0.01 | 0.82 | 0.416 | 0.891 |
| sTREM-1 | -0.00 | 0.03 | -0.06 | 0.955 | 0.985 |
| MCP-1 | 0.04 | 0.07 | 0.62 | 0.541 | 0.985 |
| IL-18 | -0.01 | 0.03 | -0.53 | 0.598 | 0.985 |
| β-NGF | 0.02 | 0.28 | 0.09 | 0.929 | 0.985 |
| α-synuclein | 0.00 | 0.00 | 1.07 | 0.289 | 0.722 |
| G-CSF | 0.03 | 0.02 | 1.08 | 0.287 | 0.722 |
| IFN-α2 | -0.04 | 0.20 | -0.21 | 0.831 | 0.985 |
| IL-2 | 0.01 | 0.46 | 0.02 | 0.985 | 0.985 |
| IL-7 | 0.02 | 0.12 | 0.12 | 0.902 | 0.985 |
| IL-1RA | -0.00 | 0.03 | -0.05 | 0.963 | 0.985 |

Table S4. Spearman’s correlation rank between immunological markers and BMI, and between immunological markers and the eigenvariate of the GMV cluster in the right thalamus. Confidence intervals were generated by bootstrapping spearman’s correlation rank (number of resamples = 2000).

| Cytokine | Outcome Variable | Spearman r | 95% CI | p-value | p(FDR) |
| --- | --- | --- | --- | --- | --- |
| IL-10 | BMI | -0.12 | [-0.42, 0.21] | 0.425 | 0.765 |
| IFN-γ | BMI | -0.19 | [-0.49, 0.15] | 0.208 | 0.765 |
| IL-6 | BMI | 0.31 | [-0.05, 0.56] | 0.034 | 0.765 |
| TNF-α | BMI | -0.18 | [-0.46, 0.16] | 0.217 | 0.765 |
| CXCL10 (IP-10) | BMI | 0.08 | [-0.22, 0.35] | 0.609 | 0.796 |
| TGF-β1 (Free Active) | BMI | -0.07 | [-0.34, 0.22] | 0.642 | 0.796 |
| sTREM-2 | BMI | 0.27 | [0.01, 0.52] | 0.068 | 0.765 |
| sTREM-1 | BMI | 0.20 | [-0.14, 0.48] | 0.183 | 0.765 |
| MCP-1 | BMI | -0.10 | [-0.42, 0.23] | 0.505 | 0.765 |
| IL-18 | BMI | 0.09 | [-0.21, 0.34] | 0.557 | 0.765 |
| BDNF | BMI | 0.06 | [-0.25, 0.36] | 0.714 | 0.826 |
| VEGF | BMI | -0.03 | [-0.32, 0.28] | 0.847 | 0.866 |
| Β-NGF | BMI | -0.22 | [-0.47, 0.06] | 0.137 | 0.765 |
| sRAGE | BMI | -0.11 | [-0.44, 0.20] | 0.463 | 0.765 |
| CX3CL1 | BMI | -0.06 | [-0.39, 0.22] | 0.669 | 0.796 |
| α-synuclein | BMI | -0.11 | [-0.43, 0.18] | 0.465 | 0.765 |
| G-CSF | BMI | -0.01 | [-0.35, 0.30] | 0.968 | 0.968 |
| IFN-α2 | BMI | -0.12 | [-0.44, 0.21] | 0.401 | 0.765 |
| IL-2 | BMI | -0.04 | [-0.34, 0.26] | 0.813 | 0.866 |
| IL-7 | BMI | -0.03 | [-0.36, 0.28] | 0.830 | 0.866 |
| IL-1RA | BMI | -0.07 | [-0.36, 0.23] | 0.650 | 0.796 |
| CXCL8 (IL-8) | BMI | -0.12 | [-0.45, 0.20] | 0.413 | 0.765 |
| IL-10 | VOI_Thalamus | 0.18 | [-0.13, 0.44] | 0.228 | 0.765 |
| IFN-γ | VOI_Thalamus | 0.16 | [-0.14, 0.43] | 0.292 | 0.765 |
| IL-6 | VOI_Thalamus | -0.11 | [-0.40, 0.15] | 0.449 | 0.765 |
| TNF-α | VOI_Thalamus | 0.05 | [-0.26, 0.34] | 0.746 | 0.842 |
| CXCL10 (IP-10) | VOI_Thalamus | 0.12 | [-0.20, 0.42] | 0.412 | 0.765 |
| TGF-β1 (Free Active) | VOI_Thalamus | -0.07 | [-0.37, 0.26] | 0.658 | 0.796 |
| sTREM-2 | VOI_Thalamus | 0.15 | [-0.17, 0.44] | 0.317 | 0.765 |
| sTREM-1 | VOI_Thalamus | -0.10 | [-0.35, 0.21] | 0.524 | 0.765 |
| MCP-1 | VOI_Thalamus | 0.14 | [-0.16, 0.45] | 0.348 | 0.765 |
| IL-18 | VOI_Thalamus | -0.10 | [-0.39, 0.20] | 0.496 | 0.765 |
| BDNF | VOI_Thalamus | -0.11 | [-0.42, 0.22] | 0.514 | 0.765 |
| VEGF | VOI_Thalamus | 0.04 | [-0.24, 0.32] | 0.781 | 0.859 |
| Β-NGF | VOI_Thalamus | 0.09 | [-0.16, 0.39] | 0.551 | 0.765 |
| sRAGE | VOI_Thalamus | 0.23 | [-0.04, 0.47] | 0.128 | 0.765 |
| CX3CL1 | VOI_Thalamus | 0.18 | [-0.10, 0.45] | 0.235 | 0.765 |
| α-synuclein | VOI_Thalamus | 0.16 | [-0.12, 0.46] | 0.277 | 0.765 |
| G-CSF | VOI_Thalamus | 0.25 | [-0.01, 0.50] | 0.081 | 0.765 |
| IFN-α2 | VOI_Thalamus | 0.14 | [-0.12, 0.44] | 0.355 | 0.765 |
| IL-2 | VOI_Thalamus | 0.21 | [-0.07, 0.50] | 0.145 | 0.765 |
| IL-7 | VOI_Thalamus | 0.16 | [-0.14, 0.46] | 0.274 | 0.765 |
| IL-1RA | VOI_Thalamus | 0.13 | [-0.15, 0.43] | 0.361 | 0.765 |
| CXCL8 (IL-8) | VOI_Thalamus | 0.28 | [-0.01, 0.54] | 0.050 | 0.765 |

Table S5. Results of linear regression models testing the interaction between immunological markers and cognitive status on GMV. The β coefficient for the interaction term represents the change in slope of the association between the respective marker and GMV in the cognitively normal group (MoCA ≥ 26) compared to the impaired group (MoCA < 26).

| Immunological  Marker | β (Interaction  Estimate) | Standard  Error | t-value | p-value | p(FDR) |
| --- | --- | --- | --- | --- | --- |
| IL-10 | -0.00 | 0.00 | -1.16 | 0.255 | 0.536 |
| IFN-γ | -0.00 | 0.00 | -1.45 | 0.154 | 0.536 |
| IL-6 | 0.00 | 0.00 | 0.20 | 0.845 | 0.905 |
| TNF-α | -0.00 | 0.00 | -0.95 | 0.345 | 0.536 |
| sTREM-2 | 0.00 | 0.00 | 2.08 | 0.044 | 0.327 |
| sTREM-1 | -0.00 | 0.00 | -3.54 | <0.001 | 0.015 |
| MCP-1 | 0.00 | 0.00 | 0.06 | 0.954 | 0.954 |
| IL-18 | 0.00 | 0.00 | 0.28 | 0.780 | 0.900 |
| β-NGF | -0.00 | 0.00 | -0.65 | 0.517 | 0.705 |
| α-synuclein | 0.00 | 0.00 | 1.00 | 0.324 | 0.536 |
| G-CSF | -0.00 | 0.00 | -1.23 | 0.225 | 0.536 |
| IFN-α2 | -0.00 | 0.00 | -1.22 | 0.231 | 0.536 |
| IL-2 | -0.01 | 0.01 | -1.60 | 0.118 | 0.536 |
| IL-7 | -0.00 | 0.00 | -0.57 | 0.571 | 0.714 |
| IL-1RA | -0.00 | 0.00 | -0.93 | 0.357 | 0.536 |

Table S6. Correlation coefficients between immunological markers and the thalamic GMV cluster in the group with normal cognitive functioning (Montreal Cognitive Assessment score ≥ 26 points). Confidence intervals were generated by bootstrapping spearman’s correlation rank (number of resamples = 2000).

| Immunological Marker | Spearman r | 95% CI | p-value | p(FDR) |
| --- | --- | --- | --- | --- |
| IL-10 | -0.00 | [-0.41, 0.39] | 0.990 | 0.995 |
| IFN-γ | -0.02 | [-0.40, 0.36] | 0.908 | 0.995 |
| IL-6 | -0.09 | [-0.44, 0.28] | 0.632 | 0.995 |
| TNF-α | -0.10 | [-0.49, 0.31] | 0.621 | 0.995 |
| CXCL10 (IP-10) | -0.01 | [-0.43, 0.40] | 0.957 | 0.995 |
| TGF-β1 (Free Active) | -0.10 | [-0.49, 0.31] | 0.604 | 0.995 |
| sTREM-2 | 0.36 | [-0.06, 0.66] | 0.058 | 0.562 |
| sTREM-1 | -0.38 | [-0.65, -0.02] | 0.040 | 0.562 |
| MCP-1 | 0.09 | [-0.32, 0.46] | 0.648 | 0.995 |
| IL-18 | -0.04 | [-0.43, 0.36] | 0.833 | 0.995 |
| BDNF | -0.09 | [-0.48, 0.34] | 0.649 | 0.995 |
| VEGF | -0.11 | [-0.47, 0.25] | 0.559 | 0.995 |
| β-NGF | 0.07 | [-0.32, 0.44] | 0.711 | 0.995 |
| sRAGE | 0.16 | [-0.22, 0.50] | 0.410 | 0.995 |
| CX3CL1 | 0.03 | [-0.38, 0.43] | 0.878 | 0.995 |
| α-synuclein | 0.33 | [-0.05, 0.62] | 0.077 | 0.562 |
| G-CSF | 0.04 | [-0.37, 0.45] | 0.818 | 0.995 |
| IFN-α2 | -0.12 | [-0.47, 0.29] | 0.535 | 0.995 |
| IL-2 | -0.10 | [-0.47, 0.30] | 0.607 | 0.995 |
| IL-7 | -0.01 | [-0.38, 0.37] | 0.954 | 0.995 |
| IL-1RA | 0.00 | [-0.39, 0.40] | 0.995 | 0.995 |
| CXCL8 (IL-8) | 0.03 | [-0.36, 0.41] | 0.879 | 0.995 |

Table S7. Correlation coefficients between immunological markers and the thalamic cluster in the group with impaired cognitive functioning (Montreal Cognitive Assessment score < 26 points). Confidence intervals were generated by bootstrapping spearman’s correlation rank (number of resamples = 2000).

| Immunological Marker | Spearman r | 95% CI | p-value | p(FDR) |
| --- | --- | --- | --- | --- |
| IL-10 | 0.45 | [0.04, 0.74] | 0.057 | 0.211 |
| IFN-γ | 0.50 | [0.06, 0.79] | 0.031 | 0.148 |
| IL-6 | -0.20 | [-0.65, 0.30] | 0.418 | 0.614 |
| TNF-α | 0.32 | [-0.17, 0.71] | 0.175 | 0.385 |
| CXCL10 (IP-10) | 0.38 | [-0.08, 0.75] | 0.109 | 0.303 |
| TGF-β1 (Free Active) | -0.05 | [-0.59, 0.49] | 0.837 | 0.877 |
| sTREM-2 | -0.16 | [-0.58, 0.38] | 0.519 | 0.635 |
| sTREM-1 | 0.38 | [-0.12, 0.70] | 0.124 | 0.303 |
| MCP-1 | 0.17 | [-0.37, 0.61] | 0.487 | 0.635 |
| IL-18 | -0.17 | [-0.67, 0.41] | 0.509 | 0.635 |
| BDNF | -0.08 | [-0.64, 0.53] | 0.797 | 0.876 |
| VEGF | 0.28 | [-0.23, 0.72] | 0.256 | 0.412 |
| β-NGF | 0.14 | [-0.40, 0.61] | 0.582 | 0.674 |
| sRAGE | 0.30 | [-0.23, 0.72] | 0.220 | 0.412 |
| CX3CL1 | 0.38 | [-0.12, 0.83] | 0.119 | 0.303 |
| α-synuclein | -0.04 | [-0.61, 0.49] | 0.883 | 0.883 |
| G-CSF | 0.49 | [0.09, 0.78] | 0.034 | 0.148 |
| IFN-α2 | 0.58 | [0.17, 0.84] | 0.009 | 0.064 |
| IL-2 | 0.65 | [0.24, 0.89] | 0.003 | 0.034 * |
| IL-7 | 0.27 | [-0.22, 0.67] | 0.256 | 0.412 |
| IL-1RA | 0.27 | [-0.19, 0.68] | 0.262 | 0.412 |
| CXCL8 (IL-8) | 0.71 | [0.36, 0.91] | <0.001 | 0.020 * |

Table S8. Correlation coefficients between immunological markers and BMI in the group with normal cognitive functioning (Montreal Cognitive Assessment score ≥ 26 points). Confidence intervals were generated by bootstrapping spearman’s correlation rank (number of resamples = 2000).

| Immunological Marker | Spearman r | 95% CI | p-value | p(FDR) |
| --- | --- | --- | --- | --- |
| IL-10 | -0.08 | [-0.49, 0.36] | 0.677 | 0.990 |
| IFN-γ | -0.12 | [-0.51, 0.29] | 0.537 | 0.990 |
| IL-6 | 0.15 | [-0.26, 0.53] | 0.437 | 0.990 |
| TNF-α | -0.07 | [-0.48, 0.34] | 0.705 | 0.990 |
| CXCL10 (IP-10) | 0.07 | [-0.31, 0.45] | 0.709 | 0.990 |
| TGF-β1 (Free Active) | 0.18 | [-0.15, 0.49] | 0.337 | 0.990 |
| sTREM-2 | 0.21 | [-0.21, 0.56] | 0.276 | 0.990 |
| sTREM-1 | 0.38 | [-0.02, 0.71] | 0.043 | 0.949 |
| MCP-1 | 0.03 | [-0.38, 0.45] | 0.876 | 0.994 |
| IL-18 | -0.00 | [-0.36, 0.36] | 0.980 | 0.994 |
| BDNF | 0.12 | [-0.29, 0.50] | 0.558 | 0.990 |
| VEGF | 0.24 | [-0.16, 0.58] | 0.215 | 0.990 |
| β-NGF | -0.23 | [-0.58, 0.15] | 0.224 | 0.990 |
| sRAGE | 0.00 | [-0.43, 0.40] | 0.994 | 0.994 |
| CX3CL1 | 0.05 | [-0.35, 0.42] | 0.783 | 0.994 |
| α-synuclein | 0.02 | [-0.40, 0.42] | 0.923 | 0.994 |
| G-CSF | 0.12 | [-0.30, 0.50] | 0.548 | 0.990 |
| IFN-α2 | -0.11 | [-0.47, 0.28] | 0.580 | 0.990 |
| IL-2 | 0.03 | [-0.34, 0.39] | 0.875 | 0.994 |
| IL-7 | -0.09 | [-0.48, 0.30] | 0.646 | 0.990 |
| IL-1RA | -0.07 | [-0.44, 0.32] | 0.720 | 0.990 |
| CXCL8 (IL-8) | -0.14 | [-0.58, 0.26] | 0.467 | 0.990 |

Table S9. Correlation coefficients between immunological markers and BMI in the group with impaired cognitive functioning (Montreal Cognitive Assessment score < 26 points). Confidence intervals were generated by bootstrapping spearman’s correlation rank (number of resamples = 2000).

| Immunological Marker | Spearman r | 95% CI | p-value | p(FDR) |
| --- | --- | --- | --- | --- |
| IL-10 | -0.40 | [-0.74, 0.06] | 0.089 | 0.380 |
| IFN-γ | -0.50 | [-0.86, 0.03] | 0.030 | 0.223 |
| IL-6 | 0.66 | [0.26, 0.90] | 0.003 | 0.060 . |
| TNF-α | -0.57 | [-0.89, -0.07] | 0.012 | 0.134 |
| CXCL10 (IP-10) | 0.04 | [-0.46, 0.56] | 0.867 | 0.908 |
| TGF-β1 (Free Active) | -0.34 | [-0.74, 0.19] | 0.168 | 0.520 |
| sTREM-2 | 0.31 | [-0.25, 0.77] | 0.209 | 0.520 |
| sTREM-1 | -0.12 | [-0.65, 0.48] | 0.644 | 0.749 |
| MCP-1 | -0.26 | [-0.62, 0.23] | 0.294 | 0.568 |
| IL-18 | 0.25 | [-0.31, 0.68] | 0.310 | 0.568 |
| BDNF | 0.03 | [-0.53, 0.60] | 0.916 | 0.916 |
| VEGF | -0.47 | [-0.76, 0.02] | 0.052 | 0.287 |
| β-NGF | -0.21 | [-0.60, 0.26] | 0.392 | 0.601 |
| sRAGE | -0.31 | [-0.71, 0.25] | 0.213 | 0.520 |
| CX3CL1 | -0.26 | [-0.68, 0.28] | 0.298 | 0.568 |
| α-synuclein | -0.39 | [-0.80, 0.21] | 0.104 | 0.380 |
| G-CSF | -0.20 | [-0.66, 0.28] | 0.410 | 0.601 |
| IFN-α2 | -0.19 | [-0.69, 0.35] | 0.441 | 0.607 |
| IL-2 | -0.17 | [-0.66, 0.40] | 0.485 | 0.627 |
| IL-7 | 0.10 | [-0.47, 0.69] | 0.694 | 0.763 |
| IL-1RA | -0.11 | [-0.61, 0.41] | 0.646 | 0.749 |
| CXCL8 (IL-8) | -0.23 | [-0.75, 0.33] | 0.350 | 0.592 |
